# MiceDEGdb: a knowledge base on differentially expressed mouse genes as a model object in biomedical research

**DOI:** 10.18699/vjgb-25-18

**Published:** 2025-02

**Authors:** O.A. Podkolodnaya, I.V. Chadaeva, S.V. Filonov, N.L. Podkolodnyy, D.A. Rasskazov, N.N. Tverdokhleb, K.A. Zolotareva, A.G. Bogomolov, E.Yu. Kondratyuk, D.Yu. Oshchepkov, M.P. Ponomarenko

**Affiliations:** Institute of Cytology and Genetics of the Siberian Branch of the Russian Academy of Sciences, Novosibirsk, Russia; Institute of Cytology and Genetics of the Siberian Branch of the Russian Academy of Sciences, Novosibirsk, Russia Kurchatov Genomic Center of ICG SB RAS, Novosibirsk, Russia; Institute of Cytology and Genetics of the Siberian Branch of the Russian Academy of Sciences, Novosibirsk, Russia; Institute of Cytology and Genetics of the Siberian Branch of the Russian Academy of Sciences, Novosibirsk, Russia Institute of Computational Mathematics and Mathematical Geophysics of the Siberian Branch of the Russian Academy of Sciences, Novosibirsk, Russia; Institute of Cytology and Genetics of the Siberian Branch of the Russian Academy of Sciences, Novosibirsk, Russia; Institute of Cytology and Genetics of the Siberian Branch of the Russian Academy of Sciences, Novosibirsk, Russia; Institute of Cytology and Genetics of the Siberian Branch of the Russian Academy of Sciences, Novosibirsk, Russia; Institute of Cytology and Genetics of the Siberian Branch of the Russian Academy of Sciences, Novosibirsk, Russia Novosibirsk State University, Novosibirsk, Russia; Institute of Cytology and Genetics of the Siberian Branch of the Russian Academy of Sciences, Novosibirsk, Russia Siberian Federal Scientific Centre of Agro-BioTechnologies of the Russian Academy of Sciences, Krasnoobsk, Novosibirsk region, Russia; Institute of Cytology and Genetics of the Siberian Branch of the Russian Academy of Sciences, Novosibirsk, Russia Kurchatov Genomic Center of ICG SB RAS, Novosibirsk, Russia Novosibirsk State University, Novosibirsk, Russia; Institute of Cytology and Genetics of the Siberian Branch of the Russian Academy of Sciences, Novosibirsk, Russia Kurchatov Genomic Center of ICG SB RAS, Novosibirsk, Russia

**Keywords:** knowledge base, DEG, mouse Mus musculus, mouse models of disease, age frustration, infectious diseases, circadian rhythm, RNA-seq, база знаний, ДЭГ, мышь, Mus musculus, мышиные модели заболеваний, возрастные расстройства, инфекционные заболевания, циркадный ритм, RNA-seq

## Abstract

The fundamental understanding of many biological processes that unfold in a human body has become possible due to experimental studies on animal models. The backbone of modern biomedical research is the use of mouse models for studying important pathophysiological mechanisms, assessing new therapeutic approaches and making decisions on acceptance or rejection of new candidate medicines in preclinical trials. The use of mice is advantageous because they have small size, are easy to keep and to genetically modify. Mice make up more than 90 % of the rodents used for pharmaceutical research. We present the pilot version of MiceDEGdb, a knowledge base on the genes that are differentially expressed in the mouse used as a model object in biomedical research. MiceDEGdb is a collection of published data on gene expression in mouse strains used for studying age-related diseases, such as hypertension, periodontal disease, bone fragility, renal fibrosis, smooth muscle remodeling, heart failure and circadian rhythm disorder. The pilot release of MiceDEGdb contains 21,754 DEGs representing 9,769 unique Mus musculus genes the transcription levels whereof were found as being changed in 25 RNA-seq experiments involving eight tissues – gum, bone, kidney, right ventricle, aortic arch, hippocampus, skeletal muscle and uterus – in six genetic mouse strains (C57BL/6J, Ren1cCre|ZsGreen, B6.129S7(Cg)-Polgtm1Prol/J, BPN/3J, BPH/2J and Kunming) used as models of eight human diseases – all these data were based on information in 10 original articles. MiceDEGdb is novel in that it features a curated annotation of changes in the expression levels of mouse DEGs using independent biomedical publications about same-direction changes in the expression levels of human homologs in patients with one disease or the other. In its pilot release, MiceDEGdb documented 85,092 such annotations for 318 human genes in 895 diseases, as suggest to 912 scientific articles referenced by their PubMed ID. The information contained in MiceDEGdb may be of interest to geneticists, molecular biologists, bioinformatics scientists, clinicians, pharmacologists and genetic advisors in personalized medicine. MiceDEGdb is freely available at https://www.sysbio.ru/MiceDEGdb

## Abstract (ru)

Фундаментальное понимание многих биологических процессов, происходящих в организме человека, стало возможным благодаря экспериментальным исследованиям на животных моделях. Основным стержнем современных биомедицинских исследований является использование мышиных моделей для изучения важных патофизиологических механизмов, оценки новых терапевтических подходов и принятия решений о переходе или отказе от новых кандидатов на препараты в доклинических испытаниях. Преимущество задействования мышей заключается в их небольших размерах, простых условиях содержания и относительно легкой генетической модификации. В настоящее время более 90 % грызунов, используемых для фармацевтических исследований, – мыши. В представленной работе создана пилотная версия базы знаний MiceDEGdb по дифференциально экспрессирующимся генам (ДЭГ) мыши как модельного объекта в биомедицинских исследованиях. Она представляет собой коллекцию опубликованных данных по экспрессии генов у мышей разных линий, предназначенных для изучения возрастных заболеваний: гипертонии, пародонтита, хрупкости кости, фиброза почки, ремоделирования гладких мышц, сердечной недостаточности, нарушения циркадного ритма. Пилотный выпуск базы знаний MiceDEGdb содержит 21 754 ДЭГ, представляющих 9769 уникальных генов Mus musculus, у которых изменяется уровень транскрипции в 25 экспериментах по технологии RNA-seq с использованием восьми тканей (десна, кость, почка, правый желудочек сердца, дуга аорты, гиппокамп, скелетная мышца и матка) в шести генетических линиях мышей, C57BL/6J, Ren1cCre|ZsGreen, B6.129S7(Cg)-Polgtm1Prol/J, BPN/3J, BPH/2J и Kunming, в качестве моделей восьми заболеваний человека согласно 10 оригинальным статьям. Новшеством MiceDEGdb в сравнении с другими базами данных о ДЭГ мышей является курируемая аннотация отклонений ДЭГ мыши от соответствующей нормы с помощью независимых биомедицинских публикаций о сонаправленных изменениях экспрессии гомологичных генов человека у пациентов с теми или иными заболеваниями относительно условно здоровых добровольцев. В пилотном выпуске MiceDEGdb документировано 85 092 таких аннотаций для 318 генов человека при 895 заболеваниях согласно 912 научным статьям, цитируемым с помощью их идентификаторов PubMed ID. Информационное содержание MiceDEGdb может быть интересным для генетиков, молекулярных биологов, биоинформатиков, клиницистов, фармацевтов и генетических консультантов по персонализированной медицине. База знаний MiceDEGdb находится в свободном доступе по гиперссылке: https://www.sysbio.ru/MiceDEGdb.

## Introduction

The use of animals is absolute to biomedical research aimed at studying biological processes (Lukacs et al., 1996), pathogenesis of diseases (Conti et al., 2002) and therapeutic interventions (Chuang et al., 2002) as well as assessing the safety, toxicity and carcinogenicity of candidate medicines (Segalat, 2007). At the same time, the relevance of animal disease models is established according to strict criteria of consistency between the animals’ conditions being studied and the symptoms the patients of interest are experiencing (Gryksa et al., 2023). To be able to interpret the results of observations made using animal models of human diseases, one should have not only knowledge of the processes being studied and pathophysiology, but also the ability to recognize spontaneous, background and associated conditions that may bias the results (White et al., 2016).

At present, more than 90 % of the pharmaceutical studies involves laboratory mouse strains (Vandamme, 2014). They are cheaper to keep than, for example, primates and can give birth every two months – these two qualities make them so popular among the researchers (Girard et al., 2009).

Although animal models still play an important role in assessing the efficiency and safety of new interventions in anticancer therapy, their use is often limited by genetic, molecular and physiological factors. Despite successful preclinical testing, 85 % of novel medicines fail during phase 1 of clinical trials: only half of those that advance to phase 3 become licensed. The use of mice as model organisms in biomedical research is deemed to be the option of choice because of their close genetic and physiological similarity with humans (Swindell et al., 2012) and because their genome is rather easy to manipulate (Monteiro et al., 2023). The latter advantage becomes more and more relevant with the advancement of genome editing methods (Bruter et al., 2024).

This inclines the researchers to move more actively to the “humanized mouse” platform, which is a good setting to use for studying the mechanisms of physiological processes (Yong et al., 2018), for exploring the pathogenesis of infectious diseases (Yajima et al., 2008; Frias-Staheli et al., 2014; Amaladoss et al., 2015; Keng et al., 2016), autoimmune diseases (Zayoud et al., 2013; Viehmann Milam et al., 2014; Gunawan et al., 2017) and cancers (Chuprin et al., 2023; Liu L. et al., 2024), and for developing anti-cancer therapies (Petrova et al., 2022). In some cases, for example, in microbiome research, wild mice should be preferred to their laboratory conspecifics, who have long been under artificial selection for the ability to breed in cages and on an ad libitum diet, which may bias the results (Hild et al., 2021).

Finally, the life sciences of the post-genome era increasingly thrive on the so-called Big Data about the differential gene expression in certain mouse tissues in the norm and pathology. The multidimensionality of Big Data requires for them to be systematized, analyzed and searched for patterns using bioinformatics methods (Liu B et al., 2024). Two sources of information become critically important for post-genome medicine and pharmacology: 1) clinical data on patients vs. unaffected volunteers and 2) experimental data on animals used as models of human diseases (Krause et al., 2023), which calls for the need of data processing resources to integrate data coming from these sources.

In one of our previous works (Chadaeva et al., 2023), we reported RatDEGdb, a freely available knowledge base on the genes that are differentially expressed in the rat used as a model object in biomedical research. As a logical step in expanding the capabilities of this series of information resources, we created MiceDEGdb, a knowledge base on DEGs in mouse strains developed in a range of scientific organizations and used as biomedical models. MiceDEGdb is freely available at https://www.sysbio.ru/MiceDEGdb

## Materials and methods

Searching PubMed for information on differentially expressed mouse genes. The experimentally identified genes that are differentially expressed in several laboratory mouse strains used as biomedical disease models were taken as published in the original articles that we found by querying [“mice” “RNA-Seq” “disease”] in PubMed (Lu, 2011).

**Fig. 1.**
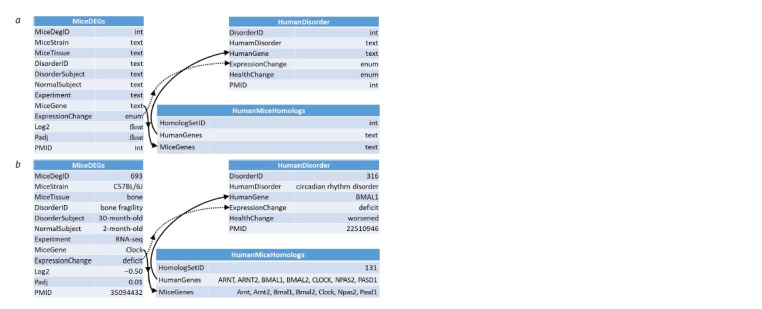
MiceDEGdb, a knowledge base on the genes that are differentially expressed in the mouse used as a model object in biomedical research into human diseases. a – flow chart; b – sample entries. Designations: MiceDEGs, HumanDisorder and HumanMiceHomologs are three unique relational tables in MiceDEGdb. In each of these tables, the left column contains the name of fields, such as “mouse gene”, “mouse strain”, “tissue”, “disease” and “the identifier of the article, to which the experimental data belong, in PubMed” (Lu, 2011); the right column is for the types of data, such as integer (int), real number (float), binary (enum) or string (text). Arrows point to relational references (solid arrows) between experimental data on the DEGs in the mouse used as a biomedical model of a particular human disease on the one hand and, on the other hand, the same-direction changes (dotted arrows) in the expression levels of human genes homologous to the DEGs in people suffering from one disease or the other – all these data were based on information in original articles found in PubMed (Lu, 2011) and referenced accordingly.

MiceDEGdb. MiceDEGdb includes three tables (see the schema in Figure 1). The mouse DEG information found as described in the previous subsection was put into a relational table, MiceDEGs. Next, we copied the relational table HumanDisorder from the one of our previous developments, Human_SNP_TATAdb database (Filonov et al., 2023); the table is explained at the bottom right of Figure 1.

Further, we linked MiceDEGs and HumanDisorder using the MiceDEGdb’s unique relational table HumanMiceHomolog based on the entries in the “Paralogs” section of GeneCards, a freely available database (Stelzer et al., 2016). Finally, we conversed MiceDEGs, HumanMiceHomolog, HumanDisorder and the links between them (the links are pointed to by arrows in Figure 1) into MiceDEGdb, which is freely available at https://www.sysbio.ru/MiceDEGdb, using MariaDB 10.2.12 (MariaDB Corp AB, Finland), a freely available open-source database management system (DBMS).

**Fig. 2.**
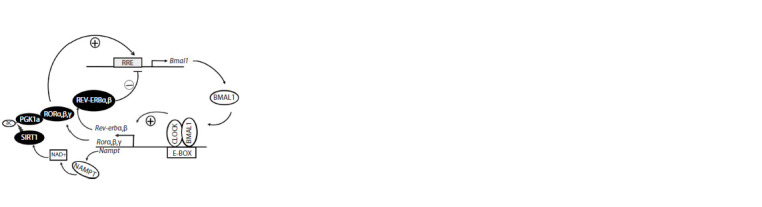
Gene regulatory network associated with the effect of the deacetylase SIRT1 on activation of Bmal1 transcription and inhibition of the CLOCK/ BMAL1 function. The oscillating feedback loop that increases Bmal1 expression and the expression of genes targeted by the transcription factor CLOCK/BMAL1 is factored in by the computational model of age-related changes in the function of the core circadian oscillator (Podkolodnyy et al., 2016): a point worth making.

**Fig. 3.**
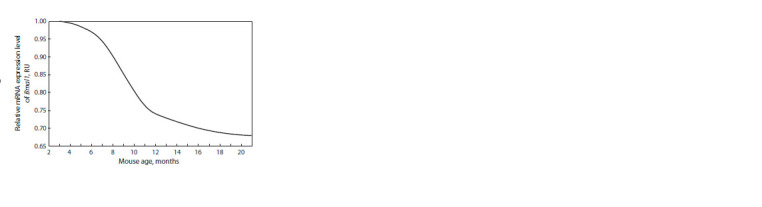
Results for the in silico modeling of changes in Bmal1 mRNA concentrations in mice using a computational model by Podkolodnyy and the co-workers (2016): the concentration levels decrease as the mice grow older.

A model assessing the effect of circadian rhythm disorder on human health. Estimates of age-related changes in the expression levels of mammalian core circadian genes were obtained using a computational model explained and validated elsewhere (Podkolodnyy et al., 2016). The outstanding feature of this model is that the interactions between the core circadian oscillator and the NAD+/SIRT1 pathway are taken into account through the use of the following modules: 1) a pathway associated with SIRT1-promoted acetylation and degradation of the PER2 protein; 2) a gene regulatory network associated with the effect of the deacetylase Sirt1 on the transcription of the mouse gene Bmal1 and inhibition of the CLOCK/BMAL1 function associated with the E-BOX through histone deacetylation (Fig. 2); 3) a pathway associated with the effect of Sirt1 on the rate at which CLOCK/BMAL1 unbinds from the E-BOX; and 4) the Nampt/NAD+/Sirt1 pathway.

In our model, the mechanism of transcriptional regulation of the Nampt gene depends on the presence of three copies of the E-BOX in its promoter, similarly to the mechanism of regulation of the Per1, Per2 and Cry1 genes in a model of the core circadian oscillator by J.K. Kim and D.B. Forger (2012).

## Results

Mouse DEGs as biomedical models of age-related diseases

Figure 3 shows the results for the in silico modeling of changes in Bmal1 mRNA concentrations in mice using a computational model by N.L. Podkolodnyy and the co-workers (2016): the concentration levels decrease as the mice grow older

To verify the results of the in silico modeling of age-related changes to the core circadian oscillator, we searched PubMed and found 10 original articles with relevance to the matter (see the Table). They presented the results of 25 RNA-Seq experiments with eight tissues (gum, bone, kidney, right ventricle, aortic arch, hippocampus, skeletal muscle and uterus) in six genetic mouse strains (C57BL/6J, Ren1cCre|ZsGreen, B6.129S7(Cg)-Polgtm1Prol/J, BPN/3J, BPH/2J and Kunming) used as models of eight human age-related diseases, including arterial hypertension, periodontal disease, bone fragility, renal fibrosis, smooth muscle remodeling, heart failure and circadian rhythm disorder. The total number of mouse DEGs was 21,754 representing 9,769 unique genes from among 22,283 annotated protein-coding genes in the reference genome GRCm38.p6 of the Mus musculus laboratory strain C57BL/6J (Sarsani et al., 2019) (see the bottom row of the Table).

**Table 1.**
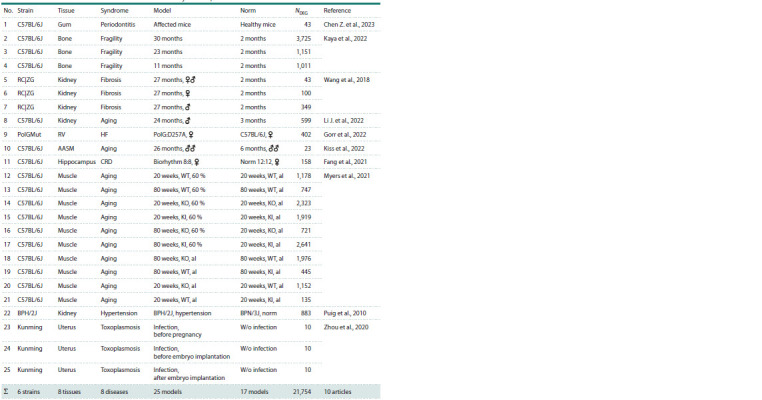
DEGs in mice used as model animals in biomedicine: revealed by RNA-Seq and documented in MiceDEGdb Notе. Mouse strain: RC|ZG – Ren1cCre|ZsGreen; PolGMut – B6.129S7(Cg)-Polgtm1Prol/J. Sex: ♀ – females; ♂ – males; ♂♂ – parabionts (surgically integrated blood systems). Tissues: RV – right ventricle; AASM – aortic arch smooth muscle. Diseases: HF – heart failure; CRD – circadian rhythm disorder; WT – wild type; KO – the Sirt1 gene knocked-out; KI – the Sirt1 gene knocked-in; al – food ad libitum.

MiceDEGdb

**Fig. 4.**
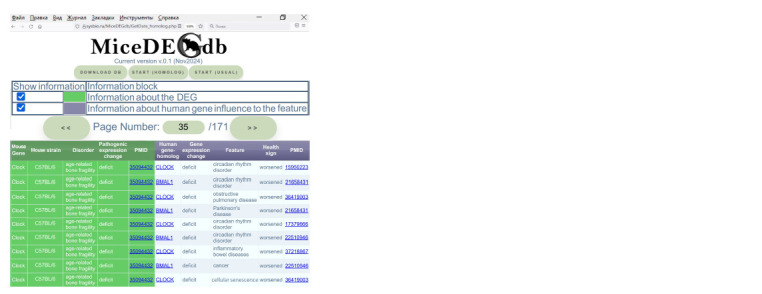
A session of MiceDEGdb, a knowledge base on the genes that are differentially expressed in the mouse used as a model object in biomedical research, for verification of the results of in silico modeling against independent experimental data.

Figure 4 shows how MiceDEGdb can be worked with. As a sample mouse gene, we took Clock. This gene was reported as being expressed at lower levels in 30-month-old male mice noted for bone fragility than in healthy males aged two months (Kaya et al., 2022).

As can be seen from Figure 4, a decrease in expression levels of a human CLOCK gene homologous to the mouse Clock gene was observed in patients with intestinal inflammation (Giebfried, Lorentz, 2023), circadian rhythm disorder (Oishi et al., 2005; Roybal et al., 2007), obstructive pulmonary disease and cellular senescence (Li L. et al., 2022), which are age-related disorders (Jacenik et al., 2019; Li Z. et al., 2021; Neba Ambe et al., 2022; Siniscalchi et al., 2024).

Additionally, the right half of Figure 4 contains the annotation that resulted from our work with experimental data on an age-related growth of bone fragility in mice concurrently with a decrease in the expression levels of the mouse gene Clock (Kaya et al., 2022) in terms of a decrease in the expression levels of BMAL1, a human paralog to Clock, according to the GeneCards database (Stelzer et al., 2016), in the following age-related human diseases: cancer (Elshazley et al., 2012), circadian rhythm disorder and Parkinson’s disease (Ding et al., 2011). This information serves to verify our in silico predictions (Fig. 3).

The pilot release of MiceDEGdb contains 85,092 such annotations for 318 human genes, changes in the expression levels of which have clinical manifestations in 895 diseases, as suggest 912 original articles referenced by their PubMed ID. The information contained in MiceDEGdb may be of interest to geneticists, molecular biologists, bioinformatics scientists, clinicians, pharmacologists and genetic advisors in personalized medicine.

MiceDEGdb is freely available at URL=https://www. sysbio.ru/MiceDEGdb

## Discussion

To show how MiceDEGdb, a knowledge base on the genes that are differentially expressed in the mouse used as a model object in biomedical research, works, we considered DEGs associated with ageing-related bone fragility in C57BL/6 mice aged from 2 to 23 and 30 months (Kaya et al., 2022).

Our attention was drawn to the differential expression levels of the Clock and Bmal1 genes encoding the components of the transcription factor Clock/Bmal1, one of the central components of the mammalian circadian oscillator, because the circadian clock system is known to be a factor of bone health (Swanson et al., 2018). Mice with the Clock gene knocked out show a reduction in bone density (Yuan et al., 2017). Mice with the Bmal1 gene knocked out are noticed to have a reduction in bone weight and density (Chen G. et al., 2020, Kikyo et al., 2024). Bmal1 regulates osteoclast differentiation and bone resorption through direct and indirect mechanisms (Chen G. et al., 2020).

The measures of differential expression of the Clock and Bmal1 genes in the C57BL/6 mice were significantly lower in the older than the younger group (Kaya et al., 2022) (Fig. 4, referenced by PubMed ID = 35094432).

As is known, aging is accompanied by circadian rhythm disorder, which coordinates virtually every process in living organisms, including bone tissue modeling and remodeling. This received further support from the results of our computational modeling which showed, in particular, that some parameters of the circadian rhythm and the expression levels of the circadian oscillator components substantially change with age (Podkolodnyy et al., 2016).

We searched PubMed for publications about same-direction changes in the expression levels of the mouse gene Clock and the human gene Bmal1 in patients with various diseases. Note that that the as-published decrease in the expression levels of these genes is typical of age-related human pathologies, such as cancer, inflammation, neurodegenerative diseases, diabetes, circadian rhythm disorder and misregulated cellular senescence (Fig. 4). The MiceDEGdb outputs of analysis of DEGs associated with the aging-related bone fragility showed that interpreting DEGs with the use of additional information in scientific publications and the results of mathematical modeling gives quite a harmonized view of age-related changes.

Finally, MiceDEGdb as a knowledge base on the mouse used as a model of human diseases is a logical step in expanding the family of databases on animal DEGs created and used for biomedical and pharmaceutical purposes. MiceDEGdb is, in a way, “sequel” to RatDEGdb (Chadaeva et al., 2023) on the ISIAH and OXYS rats, unique strains that have been developed at the Institute of Cytology and Genetics of the Siberian Branch of the Russian Academy of Sciences (Novosibirsk, Russia) and that represent genetic models of arterial hypertension and premature aging, respectively, as well as related diseases.

## Conclusion

The MiceDEGdb knowledge base is a collection of experimental data and a toolbox for interactive analysis as part of the genomic studies in the mouse used as a model object in biomedical research

The existing medical databases focus on the human genome (Sun et al., 2022), and so MiceDEGdb, which holds data on the mouse as the most frequently used laboratory animal in biomedical and pharmaceutical research, should be a valuable add-on to them

We are planning to keep updating MiceDEGdb with the main focus on the mouse gene expression data coming from the Institute of Cytology and Genetics of the Siberian Branch of the Russian Academy of Sciences (Novosibirsk, Russia). The MiceDEGdb interface (Fig. 4) will be improved following identification, accumulation and systematization of the most trending search queries.

## Conflict of interest

The authors declare no conflict of interest.
